# Association of Tumor Necrosis Factor-Alpha (TNF-α) rs1800629 Polymorphism in Chronic Kidney Disease

**DOI:** 10.7759/cureus.60332

**Published:** 2024-05-15

**Authors:** Subhashini Lanka, Vijaya Rachel K., Anuradha Arji, Riya Raju, Tarun Kumar Suvvari, Mahek Thakwani, Yarrabathina Laxmi supriya, Bharath Chandra Meenavilli, Sai Krishna Ravuru, Nagarjuna Sivaraj

**Affiliations:** 1 Biochemistry, Gandhi Institute of Technology and Management (GITAM) (Deemed to Be University), Visakhapatnam, IND; 2 Multidisciplinary Research Unit, Andhra Medical College, Visakhapatnam, IND; 3 Internal Medicine, Maharajah's Institute of Medical Sciences, Vizianagaram, IND; 4 General Medicine, Rangaraya Medical College, Kakinada, IND; 5 Research, Squad Medicine and Research (SMR), Visakhapatnam, IND; 6 General Practice, Mediciti, Hyderabad, IND; 7 Medicine, Gandhi Medical College, Secunderabad, IND; 8 General Medicine, Great Eastern Medical School and Hospital, Srikakulam, IND; 9 Medicine, Great Eastern Medical School and Hospital, Srikakulam, IND; 10 Research and Development, Great Eastern Medical School and Hospital, Srikakulam, IND

**Keywords:** chronic kidney disease, genetics, rs1800629, tnf-α, ckd, gene, polymorphism

## Abstract

Background

Chronic kidney disease (CKD) is characterized by progressive loss of kidney function. Tumor necrosis factor-alpha (TNF-α) is a cytokine implicated in inflammatory processes, including those affecting the kidneys. Although this association is not yet comprehensible, a tie-up between renal disease and markers of inflammation - interleukin-6 (IL-6), preceded by TNF-α - is eminent. However, a pause in research is evident concerning the TNF-α gene with kidney disease in the inhabitants of India. So, this study investigates the association between TNF-α rs1800629 polymorphism and CKD.

Methodology

A prospective case-control study was conducted in Andhra Pradesh for over three years. A total of 579 patients participated in the study. These were divided into premature, late-stage CKD, and control groups. The amplification refractory mutation system (ARMS)-polymerase chain reaction (PCR) was used, and biochemical investigations and genotyping were carried out for the study participants. Hardy-Weinberg expected frequencies (HWE) with chi-square test was used for detecting allele and genotype frequencies. The association between TNF-α (-308 G/A, rs1800629) and CKD was assessed using odds ratios (ORs) with 95% confidence intervals (CIs).

Results

We found a higher prevalence of CKD among males (*n *= 301, 52%) compared to females (*n *= 278, 48%). Both male and female participants diagnosed with CKD exhibited significantly elevated blood urea and serum creatinine levels compared to the control group, indicating impaired kidney function. Furthermore, these markers were generally higher in the late-stage CKD group compared to the early-stage group, suggesting a progressive decline in kidney function as the disease worsens. The homozygous genotype GG was more prevalent in late-stage CKD patients compared to both early-stage CKD patients and controls. Further, the heterozygous genotype GA was more frequent in the early-stage CKD group compared to the late-stage group. The homozygous genotype AA also showed a higher prevalence in the early-stage CKD group compared to the late-stage group. The G/G genotype and the G allele (rs1800629) were significantly associated with susceptibility to CKD (P<0.005).

Conclusions

Our study reported the TNF-α rs1800629 polymorphism and CKD risk in a South Indian population. G/G genotype and the G allele (rs1800629) were significantly associated with the risk of CKD. However, further research with larger sample sizes is warranted to confirm these observations and elucidate the underlying mechanisms by which TNF-α might influence CKD risk.

## Introduction

Chronic kidney disease (CKD) is a significant global health concern in the twenty-first century. CKD is estimated to be a major contributing factor, ranking eighth leading cause of death globally according to the 2015 Global Burden of Disease Study (GBD) study [1,2]. The rising prevalence of risk factors like diabetes and obesity is associated with an increased incidence of CKD. At present, it is trusted that adjuvant risk factors depict a considerable genetic component to CKD. In recent times, Genome-Wide Association Studies (GWAS) have recognized atypical gene variations connected with kidney disease succession and anatomy [3]. Literature quoted that a few single nucleotide polymorphisms (SNPs) are situated in genes that code for polypeptide molecules whose conversion might be a responsive agent of CKD [4]. A study by Köttgen et al. proved the interrelation of the UMOD gene with CKD. They quoted that the mutations on the gene are conducted with rare autosomal dominant tubulointerstitial disease, which contributes to the progression of CKD [5].

In the literature, the UMOD gene, GPX1, GSTO, KL, MGP, and TNF-α genes and their polymorphisms were found to have a clear association with CKD [6,7]. End-stage renal disease (ESRD) along with the disease of the kidney is marked by upgraded levels of pro-inflammatory cytokines and inflammatory markers. Polymorphisms located in lymphokine genes might impact gene rephrasing and cytokine exudation which thereby harmonize the risk of succession of CKD [8]. Previous studies have reported elevated levels of cytokines in individuals with kidney diseases [9]. Cytokines are signaling molecules primarily produced by immune cells like T cells, monocytes, and macrophages. However, research suggests that resident kidney cells, including dendritic cells, endothelial cells, mesangial cells, and tubular epithelial cells, also contribute to cytokine production. TNF-α is implicated in the recruitment of monocyte macrophages and the glomerular filtration rate (GFR) decreased rate by modulating hemodynamic variations and endothelial permeability patterns [9]. As per the literature, persons with type 2 diabetes have higher serum levels (three to four times) of TNF-α than nondiabetic patients [10]. The above polymorphism is also associated with cancers, including hepatopancreatic biliary cancers and adult acute B-cell neoplasms [11,12]. 

TNF-α is located on chromosome 6q 21.3 inside the major histocompatibility complex of the human genome [11]. Several SNPs are spotted in the locus of the gene and the promotor region, which are thought to influence transcription. The SNPs at position TNF-α (-238 G/A, rs361525) and TNF-α (-308 G/A, rs1800629) are known to influence gene expression, which is linked to various infections and autoimmune diseases and the lack of association is noted [12]. So, we aimed to investigate the association between TNF-α rs1800629 polymorphism and susceptibility to CKD.

## Materials and methods

A prospective case-control study was carried out for three years (2020-2023), and a total of 579 patients were recruited in the study. Diagnosis of damage to the kidney was made and categorized into early-stage CKD (stages 1, 2, 3a, and 3b) and late-stage CKD (stages 4 and 5) as per the National Kidney Foundation (NKF) and the Dialysis Outcomes Quality Initiative (DOQI) advisory board, which approved the clinical practice guidelines to define chronic kidney disease and stage CKD [13]. The inclusion criteria included a confirmed diagnosis of CKD and willingness to participate in the study, while individuals with other major chronic illnesses were excluded. This study was approved by the Institutional Ethical Committee (IEC) of Great Eastern Medical College and Hospital, Srikakulam, India (Reference No. 96/IEC/GEMS&H/2020).

For conducting the genotyping assay, fresh blood samples from the study participants were pooled into ethylenediaminetetraacetic acid (EDTA)-containing tubes and stored in the refrigerator. For extracting DNA, the samples were thawed, and the DNA was extracted from blood leukocytes by Miller’s salting-out method [14]. The gene polymorphism was recognized by the amplification refractory mutation system (ARMS)-polymerase chain reaction (PCR), and the gene amplification of TNF-α (-308 G/A, rs1800629) was mounted in a thermal cycler, Esco Swift Max Pro, by forward (5’-AGG CAA TAG GTT TTG AGG GCC AT-3’) and reverse (5’-TCC TCC CTG CTC CGA TTC CG-3’) primers procured from from a company in the United States, to detect the -308 changes. Both primers consist of a single nucleotide mismatch near the array site.

The PCR reaction mixture contained a master mix of 12.5 μL with 0.1 μg/μL of DNA, 3 mM of primer, 0.025 U/μL of enzyme, 1.25 μL of buffer 1X, 2.5 mM of MgCl_2_, and 2.5 mM deoxynucleoside triphosphate (dNTP). The conditions of amplification were as follows: denaturation at 94 °C for four minutes, annealing at 60 °C for 30 seconds, followed by 72 °C for two minutes for extension, which gave 107 bp fragments. The resulting DNA was analyzed on a 6% gel, which was stained with silver nitrate. The amplified fragments were then incubated with 3 U of restriction enzymes (MspI and NcoI ) for one hour at 37 °C. In the end, the PCR products were run on 6% polyacrylamide gels for identification of genotype. For the TNF-α (-308 G/A, rs1800629), 87 and 20 bp fragments constituted the genotype (G/G), 107, 87, and 20 bp amount to (G/A) heterozygote, and 107 bp fragments corresponded to homozygote (A/A).

Data were analyzed using EXCEL 2019 and IBM SPSS Statistics for Windows, Version 22.0 (IBM Corp., Armonk, NY). Hardy-Weinberg expected frequencies (HWE) with chi-square test was used for detecting allele and genotype frequencies. The association between TNF-α (-308 G/A, rs1800629) and CKD was assessed using odds ratios (ORs) with 95% confidence intervals (CIs).

## Results

A total of 579 participants were enrolled in this study. CKD was diagnosed at baseline. Among these participants, 220 (38%) were classified as having early-stage CKD, 210 (36.2%) with late-stage CKD, and 149 (25.7%) served as control subjects without CKD. The clinical parameters of the study subjects are described in Table 1. 

**Table 1:** Clinical parameters of the study subjects.

Parameters		Early-stage CKD	Late-stage CKD	Control
Age (years), mean ± standard deviation (SD)		53.4 ± 8.2	51.9 ± 8.7	52.1 ± 6.4
Male, *n* (%)		112 (19.3%)	110 (19%)	79 (13.6%)
Female, *n* (%)		108 (18.6%)	100 (17.2%)	70 (12%)
Blood urea (mean)	Male	52 mg/dL	76 mg/dL	15 mg/dL
	Female	50 mg/dL	68 mg/dL	12mg/dL
Serum creatinine (mean)	Male	32 mg/dL	40 mg/dL	1 mg/dL
	Female	28 mg/dL	34 mg/dL	0.9 mg/dL

There were no significant differences in average age across the early-stage CKD, late-stage CKD, and control groups. However, the CKD groups (early stage and late stage combined) exhibited a slightly higher prevalence of males compared to females. Importantly, both male and female patients with CKD showed significantly higher blood urea and serum creatinine levels compared to the control group, indicating impaired kidney function. Notably, these markers were generally higher in the late-stage CKD group compared to the early stage, suggesting a decline in kidney function as the disease progresses.

Allele frequency of TNF-α (-308 G/A, rs1800629) was assessed among the study population. The distribution of alleles among cases and controls is depicted in Table 2. The correlation of CKD with TNF-α is depicted in Table 3.

**Table 2:** TNF-α (-308 G/A, rs1800629) allele frequency and distribution of alleles among cases and controls. Note: X^2^ 0.05 = 5.2; the given table value is less than X^2^ and, hence, is consistent with HWE HWE, Hardy-Weinberg expected frequencies

TNF-α (–308 G/A, rs1800629)	Genotype			Allele frequency		X^2^ value	HWE
	G/G	A/A	G/A	G	A		
Early-stage CKD (*n *= 220)	156	20	44	356 (61%)	84 (14%)	0.20	0.19
Late-stage CKD (*n *= 210)	160	12	38	358 (61%)	62 (10%)	0.11	0.14
Without CKD (*n *= 149)	124	13	12	260 (45%)	38 (6%)	-	-

**Table 3:** Correlation of CKD with TNF-α (–308 G/A, rs1800629). *Statistically significant. CKD, chronic kidney disease; TNF-α, tumor necrosis factor-alpha; OR, odds ratio; CI, confidence interval

TNF-α (–308 G/A, rs1800629)	Early-stage CKD (*n *= 220)	Late-stage CKD (*n *= 210)	Without CKD (*n *= 149)	OR (95% CI)	*P*-value
Genotype					
G/G	156	160	124	0.78 (early-stage CKD and control); 0.52 (late-stage CKD and control)	0. 003* 0.004*
G/A	44	38	12		
A/A	20	12	13		

**Figure 1:**
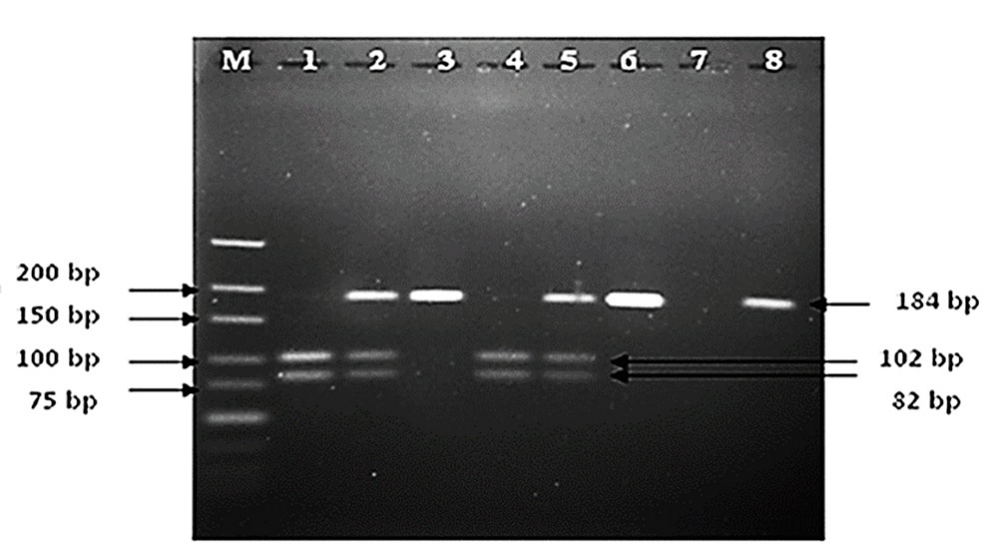
TNF-α (–308 G/A, rs1800629) band pattern. Lane M is the ladder, Lanes 1 and 4 represent the G/G genotype, Lanes 2 and 5 represent the G/A genotype, and Lanes 3 and 6 represent the A/A genotype. TNF-α, tumor necrosis factor-alpha

We also compared genotype distribution among cases and controls. Homozygous genotype GG was predominant in patients with late-stage CKD compared to early-stage and control subjects. Heterozygous genotype GA was dominant in early-stage CKD than in late-stage CKD. Another homozygous genotype AA was predominant in early-stage CKD than late-stage CKD. The study results found that the G/G genotype and the G allele (rs1800629) were significantly associated with the risk of CKD. Therefore, the presence of TNF-α (-308 G/A, rs1800629) is shown to be a strong risk factor for CKD. Figure 1 depicts the band patterns of TNF-α (-308 G/A, rs1800629) (Lane M is the ladder, Lanes 1 and 4 represent the G/G genotype, Lanes 2 and 5 represent the G/A genotype, and Lanes 3 and 6 represent the A/A genotype).

## Discussion

TNF-α gene SNPs relative to the transcription site are shown to influence gene expression and are linked to various infectious and autoimmune diseases [15]. Moreover, cytokines trigger organ injury, and increased levels of TNF-α are thought to improve adverse clinical outcomes in kidney disease patients [16]. Furthermore, polymorphisms in the TNF-α promotor region are known to affect transcriptional activity and show adverse clinical outcomes in critically ill patients [17-19]. Hence, in the present study, the association of TNF-α (-308 G/A, rs1800629) with susceptibility to CKD in the South Indian population was assessed.

The elevation of TNF-α levels in patients with CKD and the underlying mechanism causing this increase are not clear. Several SNPs present in the promotor region of TNF-α affect the expression, and thus, -308 G/A (rs1800629) was selected for the study. The status of this SNP in patients with CKD was found to have a strong association between the TNF-α (-308 G/A, rs1800629) and CKD. To our knowledge, this is the first study to investigate the association between the TNF-α rs1800629 polymorphism (-308 G/A) and CKD in a South Indian population. Prior studies on several other populations have identified a significant correlation between this SNP and CKD risk [20,21]. The present findings add to the growing body of evidence, suggesting a potential role for TNF-α in CKD susceptibility within Asian populations.

A link between TNF-α signaling and endoplasmic reticulum stress has been reported in the context of CKD-dependent vascular calcification [20]. While the augmentation of these signaling pathways and their role in TNF-α-mediated vascular calcification remains an area of active investigation, the study suggests a potential link to increased phosphate absorption. Specifically, the researchers found that the type III phosphate transporter and Pit-1, a key player in the PERK-eIF2α-ATF4-CHOP pathway, are crucial modulators of vesicle calcification [20]. Additionally, the study noted that C/EBP homologous protein may significantly enhance phosphate uptake by upregulating Pit-1 expression.

It is important to note that in the later stages of CKD, the renal system's capacity for phosphorus excretion is markedly reduced, leading to elevated serum phosphorus levels alongside increased calcium and calcium-phosphate products. This contributes to the progression of vascular calcification observed in these patients [22]. The potential link between TNF-α levels and increased phosphorus warrants further investigation in future studies.

While the present study sheds light on the potential association between TNF-α rs1800629 and susceptibility to CKD in a South Indian population, a few limitations exist. The small sample size might limit the generalizability. Additionally, environmental factors and their interactions with the polymorphism were not evaluated. Finally, the observed associations needed to be confirmed in larger studies and explored in other populations to assess their broader applicability.

## Conclusions

Our study reported the TNF-α rs1800629 polymorphism and CKD risk in a South Indian population. G/G genotype and the G allele (rs1800629) were significantly associated with the risk of CKD. However, further research with larger sample sizes is warranted to confirm these observations and elucidate the underlying mechanisms by which TNF-α might influence CKD risk. Additionally, investigating potential interactions between the polymorphism and environmental factors could provide a more comprehensive understanding of CKD development in this population. Our study provides a foundation for future studies about the development of genetic screening tools to identify individuals at higher risk for CKD, potentially enabling earlier intervention and personalized treatment strategies.
